# Cross-cultural adaptation and psychometric validation of the Chinese version of career success in nursing scale (CSNS)

**DOI:** 10.1186/s12912-023-01415-5

**Published:** 2023-07-28

**Authors:** Yuan-Yuan Cui, Xia Zhong, Li-Ying Wen, Xing-Yu Chen, Xing-Hua Bai

**Affiliations:** 1 Department of Radiation Oncology, The First Hospital of China Medical University, Shenyang, China; 2 School of Nursing, Dalian University, Dalian, China

**Keywords:** Career success, Cross-cultural adaptation, Psychometrics, Reliability, Validity

## Abstract

**Objective:**

To translate the career success in nursing scale (CSNS) into Chinese and evaluate its psychometric properties.

**Background:**

A lower sense of career success seriously affects the enthusiasm of nurses and increases their turnover rate. Therefore, an accurate assessment of the career success level of nurses is necessary. However, China does not have a professional tool for assessing the career success of nurses.

**Methods:**

The stratified sampling method was used to recruit participants from 22 hospitals of different grades in 5 cities in China. A total of 650 and 348 subjects were selected for item analysis and reliability and validity tests, respectively, of the translated initial scale.

**Results:**

The Chinese version of the CSNS (C-CSNS) with 33 items had good psychometric properties. Cronbach’s α was 0.960, split-half reliability was 0.893, and ICC within two weeks was 0.981. Exploratory factor analysis extracted 5 common factors that explained 63.73% of the total variance, and confirmatory factor analysis supported acceptable construct validity.

**Conclusion:**

The C-CSNS has adequate construct validity and excellent psychometric properties and can be used for accurate assessment of nurses’ career success.

**Implications for nursing management:**

A new tool that is more suitable for the Chinese hospital nursing context is available for evaluating Chinese clinical nurses’ career success. Nursing managers can formulate appropriate management strategies according to the evaluation results to assist nurses in career development planning, thereby improving their career success level.

## Introduction

Since the nursing profession has been reinstated in higher education in China, the nursing industry has changed and grown substantially over the past 20 years [1]. The professional and technical level of nurses is no longer the only way to tell if a nurse is good or bad [2]. In the nursing industry, there are many development directions for nursing staff to take to advance their careers so that they can achieve a sense of value and a higher level of career success [2, 3]. Therefore, in recent years, how to improve the career success level of nurses has become a focus of individual nurses and nurse managers, and this interest has introduced new concepts such as job satisfaction, professional satisfaction and career commitment to the discussion about career success [4].

Career success is defined by an individual’s positive psychological satisfaction and self-realization, and it is the psychological goal that practitioners constantly pursue in their work. To cultivate high-quality nursing talent, we should not only focus on strengthening nurses’ professional skills but also their psychological goals, and career success is one of the psychological mechanisms that affect the career pursuit of nurses [4, 5]. Improving career success for individuals can not only improve self-identity and relieve negative emotions but also enable individuals to have stronger work motivation in daily work, lighten burnout, and reduce work errors [6, 7]. Therefore, improving work efficiency and work quality and achieving higher career success form a virtuous circle. For hospitals, improving the level of career success can reduce the loss of nursing talent, promote the development of the nursing profession and improve the quality of medical services, which plays an important role in stabilizing the nursing talent team, improving the efficiency of nursing management, and improving the professional level and competitiveness of hospitals [8, 9].

Therefore, research on the career success of nurses is very important. An understanding of nurses’ career success needs to be established first with an adequate assessment of this segment of the population [8]. However, due to the differences in the national context and health care environment, research on the career success of nursing staff in China started relatively late and has used limited research tools. Some scholars have used the universal career success scale to assess nurses [10]. However, the special clinical operations and complex medical environment lead to significant differences in the job content and the professional environment of nurses compared to general staff. In the nursing field, in addition to performing simple basic nursing operations and some difficult specialist technical operations, nurses also need to address patient emotions, hospital management, title promotion, teaching and lecturing, specialist nurse training, and prescribing postdischarge nursing care [11–13]. General scales can hardly reflect the many characteristics of the nursing profession. Therefore, the assessment of nurses’ career success must be consistent with the clinical nursing environment.

The CSNS developed by Asghari et al. is the latest career level assessment tool for the specific occupation of nursing [14]. The scale has a comprehensive theoretical structure, is based on nursing work and the nursing career and has been well used locally. It has been shown to have good reliability and validity, is simple to understand, takes 10 min to complete and is easy to score. Mohammadzadeh et al. [15] used CSNS in a survey of emergency department nurses in eight university hospitals in Iran where it showed good utility and reliability. The results of their study suggest that nursing managers and leaders should provide a healthy work environment to help nurses increase their professional success. Previous research has also shown that hospital administrators should improve their understanding of nurses’ career success levels so that they can target their efforts to help nurses with low success levels plan for career development and take steps to improve their sense of success [5, 16]. This will also reduce nurse turnover rates and result in a more stable, highly qualified nursing workforce [5, 17].

We aimed to translate the CSNS into a Chinese version and identify its psychometric characteristics in the Chinese nurse population, with a view to providing hospital administrators with a new tool for assessing nurses’ career success that is more suitable for the Chinese nursing environment.

## Methods

### Study design and participants

From February to July 2022, two cross-sectional surveys were conducted in 22 hospitals in five cities in China. To reduce selection bias and collect as much data as possible to make the conclusions more representative, we adopted a sampling method combining stratification and convenience (that is, we first classified hospitals according to their grades as first-class hospitals, second-class hospitals, and third-class hospitals and then recruited subjects from hospitals of different levels). According to the recommendations [18, 19], the sample size of exploratory factor analysis (EFA) should be 10–20 times that of each item, and confirmatory factor analysis (CFA) requires a sample size of at least 200. Since CFA cannot use the same data set as EFA [20] but should use different data for cross-validation to ensure the scientific nature of the structural model, we recruited two samples. As the original version of the CSNS has a total of 39 items, the sample size of EFA should be 390–780, and the sample size of CFA should be more than 200. By the end of the study, a total of 650 and 348 nurses participated in the EFA and the CFA, respectively. Forty nurses were selected and tested again two weeks after the first test to calculate the test-retest reliability of the scale. The inclusion and exclusion criteria for this study are as follows: Inclusion criteria: ① Nurse practitioners who hold the Nurse Practitioner Certificate and were registered. ② Nurses who signed informed consent forms and agreed to participate in this research. Exclusion criteria: ① Nurses who were in the target survey hospital for study or practice. ② Nurses who were not on duty due to illness, maternity or personal leave during the survey period.

### Setting

#### Translation process

First, we obtained the English and Persian versions of the scale with the authorization of Asghari, the author of the original scale. We adopted the Brislin translation model and strictly follow the requirements of the cross-cultural research tool adaptation and validation guidelines for sinicization [21, 22]. In the first step, two translators with strong English ability and native Chinese speakers (a master of nursing who works in Canada and is proficient in English and an associate professor of nursing with CET-6) independently translated the original English into two Chinese versions. In the second step, the met with two translators, who were asked to compare their Chinese versions with the original scale and discuss any differences. They then negotiated and changed the differences until they agreed on a solution. In the third step, two other translators who were bilingual had not been exposed to the original scale and did not know that they were performing back translation (two M.D.s who were fluent in English and had studied in the United States) independently back-translated the Chinese version. The fourth step was to organize a second meeting with researchers and translators to compare, discuss and revise the back-translated version with the original scale until an agreement was reached.

#### Cross-cultural adaptation

In this study, the Delphi method was used to adjust the scale across cultures. We invited six senior clinical nursing experts to form an expert group (including two nursing professors with more than 30 years of nursing management experience and professional knowledge and four associate professors who had been engaged in the nursing industry for more than 15 years with rich nursing management experience) and distributed the expert consultation questionnaire compiled from the Chinese version of the scale after translation and revision. There was no horizontal relationship of mutual communication among the experts. After two rounds of correspondence and induction, a basically consistent result was formed to examine the equivalence of the scale.

### Ethics considerations

This study was approved by the Ethics Committee of the First Affiliated Hospital of China Medical University (approval number: [2021] No.505). All participants gave informed consent and voluntarily participated in this study. In addition, all information collected from participants was kept strictly confidential and used only for this study.

### Instruments

#### General information questionnaire

Including gender, age, education, hospital level, department, technical title and employment method.

#### Career success in nursing scale (CSNS)

The scale was compiled by Asghari [14] in 2020. It contains 39 items in four dimensions: expected career progression, provision of quality care, effective self-regulation and person-organization fit. It reflects the degree of career development expectations that nurses have achieved at work, the ability to complete high-quality nursing care, the ability to use effective self-monitoring and management strategies to perform well at work and achieve work-life balance, and whether the task roles given by their organizations are effectively matched to their skill set. A 5-point Likert scale is used (1= “strongly disagree”, 5= “strongly agree”), and the higher the score is, the higher the sense of career success for nurses. The Cronbach’s α coefficient of the scale is 0.93, the Cronbach’s α value of each dimension subscale is 0.83 to 0.91, and the two-week test-retest reliability is 0.90, with good reliability and validity.

#### Career success scale (CSS)

The Career Success Scale was used to evaluate the criterion-related validity. The scale was originally a self-evaluation scale developed by foreign scholar Eby [23] and includes three dimensions and 11 items. Responses are measured on a 4-point Likert scale, and the total score ranges from 11 to 44. A higher total score means a higher sense of career success. The Cronbach’s α coefficient of the Chinese version of the Career Success Scale ranges from 0.74 to 0.85.

### Pretesting

We selected 30 nurses who met the inclusion criteria via convenience sampling and sent the first draft of the survey to them. The participants were then asked if there were any incomprehensible or clinically impractical aspects about the setting and meaning of the questionnaire items to ensure its practical application.

### Data collection

Online and on-site synchronization. The C-CSNS was uploaded to an online platform, and the link was sent to the individuals who agreed to participate in the research by means of WeChat or e-mail. The researcher also provided a paper version of the questionnaire and provided it to recruited nurses who could complete it on site.

### Data analysis

Data entry and analysis were performed using SPSS 26.0. All tests were two-tailed, and the test level was two-sided α = 0.05. The specific statistical methods are as follows: quantitative data are expressed as the mean and standard deviation; qualitative data are expressed as the frequency and percentage. The item analysis of the scale used the item distribution method, critical ratio method and Spearman correlation coefficient method. Cronbach’s α coefficient, split-half reliability, and two-week test-retest intraclass correlation coefficient (ICC) were used to evaluate the reliability. Validity was evaluated with the content validity index (CVI), EFA, CFA and criterion-related validity evaluation.

## Results

### Translation results

After cross-cultural debugging, we made some appropriate modifications to the expressions of some items. At the same time, Item 24, Item 29, Item 33 and Item 38 did not conform to the Chinese medical environment and medical regulations, had little relationship with the scale and dimension themes and had semantic overlap with other items. Thus, these items were deleted, so that the scale items after cross-cultural adjustment were more appropriate for the Chinese medical environment and the job content of nurses. At this point, we tentatively obtained a preliminary C-CSNS of 35 items.

### Demographic information and characteristics of respondents

In the data collection phase, we recruited 685 and 383 nurses, for the EFA and the CFA groups, respectively. After the questionnaire quality inspection, 650 and 348 valid questionnaires were screened out, and the effective rates of the questionnaires were 94.9% and 90.9%, respectively. The general demographics and distribution characteristics of the participants are shown in Table 1.

**Table 1:** The general demographic information of the participants(n = 998)

Characteristic	n (%)	
	EFA Group (n = 650)	CFA Group (n = 348)
Gender		
Male	77(11.8)	22(6.3)
Female	573(88.2)	326(93.7)
Age		
≤ 25	125(19.3)	48(13.8)
26~35	297(45.7)	153(44.0)
36~45	179(27.5)	113(32.4)
≥ 46	49(7.5)	34(9.8)
Education status		
Technical secondary	37(5.7)	17(4.9)
College	125(19.2)	51(14.7)
Bachelor	443(68.2)	269(77.3)
Master	45(6.9)	11(3.1)
Hospital grade		
Grade I	78(12.0)	29(8.3)
Grade II	104(16.0)	33(9.5)
Grade III	468(72.0)	286(82.2)
Department		
Internal medicine	184(28.3)	100(28.7)
surgery	138(21.2)	76(21.8)
Obstetrics and Gynecology	43(6.6)	21(6.0)
Pediatrics	27(4.2)	17(5.0)
Outpatient	41(6.3)	20(5.7)
Operating room	31(4.8)	18(5.2)
Intensive care unit	47(7.2)	22(6.3)
Other departments	139(21.4)	74(21.3)
Technical titles		
Nurse	130(20.0)	50(14.4)
Senior nurse	332(51.0)	170(49.0)
Supervisor nurse	174(26.8)	120(34.5)
Associate professor/Professor of nursing	14(2.2)	8(2.3)
Employment method		
Authorized strength	128(19.7)	68(19.5)
Contract system	512(78.8)	278(80.2)
Other	10(1.5)	1(0.3)

### Item analysis

The selection results of 650 people were counted by the item distribution method, and the results showed that the selection rate of each option for the 35 items was less than 80%, indicating that each item had a good distinguishing ability. The total score of career success was sorted; the top 27% were the high group, and the bottom 27% were the low group, and the Mann‒Whitney U test was performed. The results showed that the p values were all < 0.01, indicating that the scale was able to distinguish subjects at different levels. Spearman correlation analysis was used to calculate the correlation between the score of each item and the total score of the scale. The results showed that the correlation coefficient was 0.49–0.80, and the correlation coefficients of all items were > 0.4, P < 0.01, indicating that each item of the scale was representative and independent. One of the 35 items on the translated scale was deleted, and then the Cronbach’s α coefficient for the remaining 34 items was tested. The results of the study showed that whenever an item was removed, the Cronbach’s α coefficient of the remaining items decreased, which indicated that each item in the scale contributed to the internal consistency of the overall scale. After item analysis, no further items needed to be deleted from the scale.

### Validity analysis

#### Content validity and face validity

The CVI was calculated for the scores of the scale items by experts in the second round of expert consultation. The results showed that the item of the content validity index (I-CVI) of the C-CSNS ranged from 0.83 to 1.00, and the scale of the content validity index (S-CVI) was greater than 0.9, with excellent content validity. The 30 nurses who participated in the preexperiment agreed that none of the items of the C-CSNS were difficult to understand or hard to answer, the number of items was appropriate, and it only took 5–10 min to complete on average.

#### EFA

EFA of the scale was performed using principal component analysis and Oblimin oblique rotation. To ensure the validity and adaptability of factor analysis, we first performed the KMO test and Bartlett’s test of sphericity on 650 sample data to judge whether the scale was suitable for factor analysis. The results showed that the KMO value of C-CSNS was 0.950, which exceeded the recommended value of 0.8, the approximate chi-square value of Bartlett’s test of sphericity was 16060.928, the degree of freedom was 595, and p < 0.001 reached a significant level. The above data show that there are common factors among the overall correlation matrices, which are suitable for factor analysis.

**Fig. 1:**
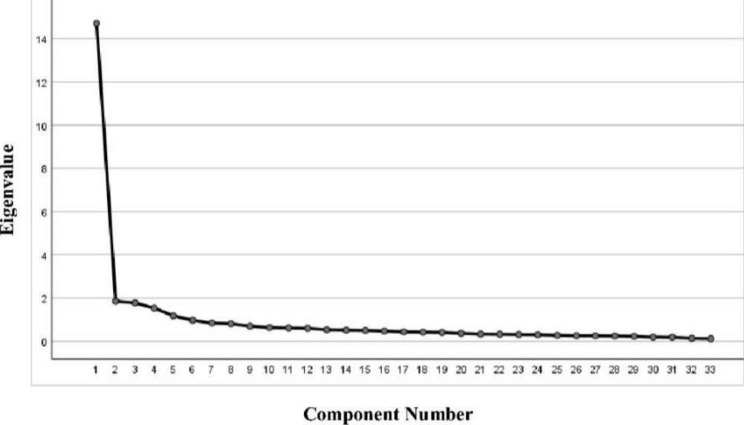
Exploratory factor analysis scree test scree plots

Factors with eigenvalues > 1 were extracted and combined with the decreasing variation of the factors in the gravel diagram (Fig. 1). Five common factors were finally extracted, with a cumulative contribution rate of 63.81%. The factor loadings of each entry were greater than 0.4, which met the standard. However, since Item 5 “I carry out my duties with interest and energy” and Item 8 “I have advanced enough in the nursing profession, and I have no intention of making progress” have load values greater than 0.4 in both factors, appearing cross-loaded, they were deleted. The five common factors extracted from the remaining 33 items explained 63.73% of the total variance. In addition, Items 1 to 15, which originally belonged to one of the topics “Expected career progression”, were split into two new factors after EFA. Items 1–7 (Item 5 deleted) belong to the same factor, and Items 9 to 15 belong to the same factor (see Table 2).

The remaining 33 items were renumbered, and new factors were named according to the common themes reflected by the items. Because Items 1–6 are related to career development and learning, this dimension is named “Career development ability”, which corresponds to the theme of “embarking on career growth” in the original development theory and reflects the behavior and ability of nurses’ professional development in clinical work. Items 7–13 are related to work attitude. Combined with the theme of “having positive personal attributes” in the original development theory, we named this dimension “positive working attitude”, which reflects nurses’ active working attitude and upward career development intention in clinical work.

**Table 2:** Exploratory factor analysis results of the revised 33-item C-CSNS (n = 650)

Original CSNS item number and description		Factor loading				
		F 1	F 2	F 3	F 4	F 5
1	My performance has a positive impact on overall assessment and outcomes of the ward.	0.065	0.075	0.087	0.196	**0.521**
2	I work in line with my conscience.	0.185	0.087	0.039	0.069	**0.584**
3	I use general knowledge such as English and the Internet to make progress in my work.	0.024	0.031	0.192	0.074	**0.706**
4	I try to make society’s attitude to nursing positive.	0.000	0.241	0.128	0.117	**0.707**
6	In the shift work, I have mastered the status of the entire department and each of my patients.	0.111	0.272	0.099	0.206	**0.426**
7	During my service, I have been able to help save lives.	0.215	0.168	0.012	0.186	**0.479**
9	To ensure that the patient is not harmed, I pay attention to the actions of doctor and my colleagues.	0.229	0.159	0.110	**0.445**	0.166
10	I have mastered my ward’s specialized equipment, such as the electroshock machine and a variety of pumps.	0.060	0.079	0.014	**0.676**	0.158
11	Before taking care, I consider all the benefits, disadvantages, and possible consequences.	0.206	0.010	0.199	**0.491**	0.012
12	I keep my nursing knowledge up to date.	0.099	0.001	0.011	**0.695**	0.135
13	In emergency situations, I can manage the patient’s clinical condition until the doctor arrives.	0.069	0.028	0.053	**0.826**	0.02
14	I transfer my knowledge and experience to new colleagues and students.	0.077	0.061	0.178	**0.632**	0.076
15	I take initiative in my work and hospital.	0.101	0.017	0.031	**0.753**	0.206
16	I respect the dignity and personality of the patient.	**0.744**	0.103	0.055	0.005	0.178
17	I feel responsible for the patients and their problems.	**0.759**	0.096	0.028	0.031	0.206
18	In clinical decision-making, I will prioritize patient’s benefits.	**0.827**	0.109	0.007	0.028	0.072
19	I take care of the patients with compassion.	**0.614**	0.233	0.053	0.011	0.112
20	When providing care, I consider the specific characteristics and needs of each patient.	**0.782**	0.135	0.019	0.139	0.147
21	I try to provide safe care to my patients.	**0.546**	0.041	0.255	0.035	0.031
22	By getting the patient’s trust, I’ll be the source of comfort for him/her.	**0.483**	0.239	0.095	0.121	0.029
23	I have the needed practical skills to do my job.	**0.631**	0.236	0.078	0.173	0.177
25	I support the rights of clients.	**0.506**	0.176	0.234	0.001	0.076
26	I start the error correction from my own self.	0.182	**0.725**	0.002	0.077	0.060
27	To take care of myself, I observe the occupational safety standards.	0.242	**0.472**	0.111	0.091	0.186
28	I care about my appearance and professional look.	0.208	**0.532**	0.136	0.131	0.243
30	I consider myself a highly regarded and valuable nurse.	0.120	**0.569**	0.078	0.055	0.068
31	I keep my composure in different working conditions.	0.036	**0.803**	0.058	0.088	0.023
32	Despite work related problems and pressures, I carry out my duties well.	0.040	**0.840**	0.030	0.004	0.006
34	I balance and coordinate between personal and business affairs.	0.157	**0.663**	0.261	0.029	0.089
35	I do my job according to job description.	0.014	0.062	**0.776**	0.031	0.061
36	If necessary, I will assist my colleagues in nursing care.	0.018	0.101	**0.816**	0.032	0.012
37	I cooperate with the authorities in planning and providing care.	0.020	0.053	**0.774**	0.097	0.040
39	I treat all the staff and doctors in the hospital respectfully.	0.110	0.074	**0.788**	0.005	0.008
Eigenvalue		14.699	1.849	1.775	1.531	1.176
Percent of total variance explained (%)		44.543	5.602	5.38	4.64	3.565
Cumulative percent of total variance explained (%)		44.543	50.145	55.526	60.166	63.73

#### CFA

**Fig. 2:**
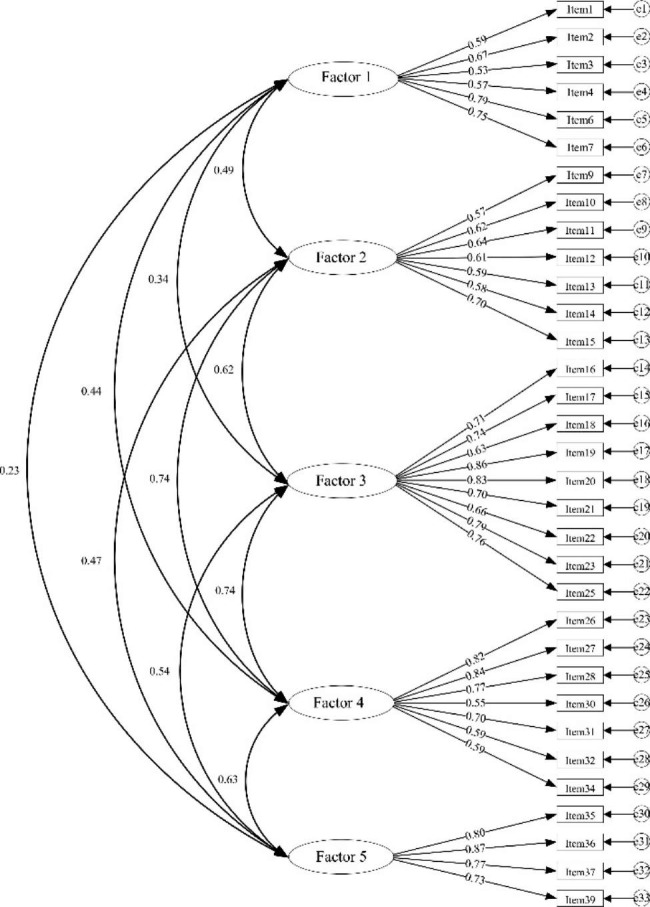
Hypothesized confirmatory factor analysis model of the C-CSNS. Factor 1: Career development ability; Factor 2: Positive working attitude; Factor 3: Providing quality care; Factor 4: Effective self-regulation; Factor 5: Person-organization fit.

Figure 2 shows the hypothesized CFA model of the C-CSNS. By comparing the degree of fit, it is found that the modified five-factor model has acceptable fitting indicators (χ^2^ = 820.904, χ^2^/*df =* 1.769, RMSEA = 0.05, SRMR = 0.05, CFI = 0.94, IFI = 0.94, TLI = 0.93) (see Table 3).

**Table 3:** Model fit indices of the CFA of the modified five-factor models

Model fit indices	χ^2^	*df*	χ^2^/*df*	RMSEA	SRMR	CFI	IFI	TLI
Reference criteria			≤ 3	≤ 0.08	≤ 0.05	≥ 0.90	≥ 0.90	≥ 0.90
Five-factor model	820.904	464	1.769	0.05	0.05	0.94	0.94	0.93

#### Criterion-related validity

Spearman correlation analysis was used to test the correlation between C-CSNS and CSS. The results showed that the total scores of the two scales were positively correlated (r = 0.641, P < 0.01), and the dimensions of the two scales were also positively correlated (r = 0.237 ~ 0.668, P < 0.01), indicating that the criterion-related validity of the C-CSNS is good.

### Reliability analysis

The Cronbach’s α of the C-CSNS is 0.960, and the Cronbach’s α of each dimension is 0.842–0.927. The split half reliability (front and back split half) of the C-CSNS is 0.893, and each dimension is 0.787 ~ 0.873. The two-week test-retest data of 40 participants showed that the test-retest reliability ICC of the C-CSNS was 0.981, and the ICC of each dimension was 0.892–0.982 (see Table 4).

**Table 4:** Reliability coefficient of the scale

Dimension	Number	Cronbach’s α	Split half reliability	test-retest reliability (ICC)
Career development ability(F1)	6	0.842	0.826	0.892
Positive working attitude(F2)	7	0.874	0.787	0.982
Providing quality care(F3)	9	0.927	0.864	0.938
Effective self-regulation(F4)	7	0.897	0.855	0.970
Person-organization fit(F5)	4	0.864	0.873	0.919
Total CSNS scale	33	0.960	0.893	0.981

### Floor/ceiling effect

Out of a total of 998 nurses in Group 1 and Group 2, no nurses (0%) achieved the lowest (33) and the highest (165) scores, demonstrating the absence of a floor/ceiling effect.

## Discussion

To the best of our knowledge, there is currently no special assessment tool in China to evaluate the self-perceived career development success of nurses. Therefore, this study is the first and has certain significance for career development research on Chinese nurses.

### Language validity

Translation is a process by which information from the source language can be transferred to the target language. The fundamental problem of using foreign assessment tools is the correct translation and implementation of a culturally appropriate tool in the target community [24]. As the translations of items may not express the same meaning in the target language [25, 26], experts have been requested to assess the restatement from a semantic point of view. Some minor revisions have been made to clarify the meaning of survey items in the Chinese medical environment, taking into account the experts’ suggestions. For example, wording arrangements were made for Item 6 (In the shift work, I have mastered the status of every patient I was responsible for), Item 7 (During my care for patients, I am able to do everything I can to help them regain their health) and Item 35 (I do my job according to the system and norms). In addition, in the cross-cultural adaptation stage, four deletions were made to remove items that did not conform to the Chinese medical environment and medical regulations, had little relationship with the scale and dimension themes and had semantic overlap with other items. These included Item 24 (I try to provide comfort and relaxation for the patient), Item 29 (I do not need to emphasize or follow up with the authorities to do my duties), Item 33 (Prior to doing my work, I prioritize them) and Item 38 (I feel responsible for the property of the hospital, such as medications and equipment). The results of the scale cross-cultural adaptation stage of this study showed that the translation of the source scale into Chinese was acceptable. As a result, excellent and culturally appropriate translation of the CSNS provides the opportunity to compare the concepts between the target and reference societies.

### Validity

Since items can be distorted during translation, the translated tool needs to be revalidated with the target population [27]. The validity of the scale was evaluated by the content validity CVI values, the criterion-related validity ICC values, and the structural validity that incorporates EFA and CFA. The results showed that the content validity CVI value and criterion-related validity ICC value of the C-CSNS were within acceptable values [28, 29].

The original scale was a four-factor model [14]. After deleting four items in the cross-cultural adjustment, we performed a comprehensive EFA on the remaining 35 items in the scale. In EFA, we found in the early stage that there were correlations between the items, whether at the theoretical level or in the correlation coefficient. Therefore, we used oblimin rotation to explore the factor structure, which also maintained consistency with the original scale development process. However, there were two items with loading values higher than 0.4 on both factors. After removing these two items, we determined five factors. Of the determined factors, *career development ability* included six items, *positive working attitude* included seven items, *providing quality care* included nine items, *effective self-regulation* included seven items and *person-organization fit* included four items. These factors jointly explained 63.7% of the total variance. The factor loading of each item in each factor was higher than 0.4. The results obtained by EFA meet the criteria that serve as the basis for these determined factors and items [30, 31].

The development process of the original scale did not involve CFA, which is a limitation. To better clarify the factor structure so that it can be properly applied to the Chinese nurse population, we performed CFA on the C-CSNS after EFA using a sample of 348 cases. The model fitting index results show that all indices of the five-factor model are within the acceptable range [32, 33]. These findings support the five-factor structure of the C-CSNS. It shows that the C-CSNS has good construct validity and can stably and accurately assess the career success levels of Chinese nurses.

### Reliability

The reliability of the scale was evaluated by internal consistency reliability Cronbach’s α coefficient, split-half reliability and two-week test-retest reliability ICC values. The results of this study showed that the Cronbach’s α of the 5-Factor C-CSNS was 0.960, and the split-half reliability was 0.893, similar to the original scale. This indicates that the C-CSNS has good internal consistency reliability among Chinese nurses [34]. In addition, the ICC of the two-week test-retest reliability of the C-CSNS was 0.981, and the ICC of each subscale was between 0.892 and 0.982, which is also similar to the original version and slightly higher than the original scale. This suggests that C-CSNS has some stability across time [35].

In conclusion, the C-CSNS has ideal reliability and stability in evaluating the career success of Chinese nurses.

### Limitations

The study also had some limitations. First, during the translation process, we did not use the original Persian version but the English version provided by the original author. Although this practice was approved by the author of the original scale, it may also have an impact on semantic understanding. Second, there is another issue to be pointed out. As a result of the novelty of this tool and the development and verification of the source tool in Persian, researchers are faced with a shortage of resources to obtain more studies in this area for a better discussion. Finally, during the localization process of this study, some items of the scale were deleted, and the number of items was different from that of the source scale. Thus, a comparison between the Chinese version and the original version cannot be conducted.

## Conclusions

This study demonstrates that the Chinese version of the CSNS has adequate construct validity and excellent psychometric properties in assessing nurses’ career success levels. The successful localization of the scale provides an easy-to-use assessment tool that is more suitable for the Chinese health care environment, nurses and nurse managers.

## Implications for nursing management

In the increasingly tense medical environment, the problem of nurse turnover and coping mechanisms has always been the focus of nursing management. To solve this problem, it is undoubtedly important to evaluate and understand nurses’ sense of gratification and success in their professional development. This study provides Chinese nursing managers with a new tool for assessing the career success of clinical nurses that is more suitable for the hospital nursing context. The C-CSNS can help nurse managers identify nurses’ low sense of career success, formulate appropriate management strategies to address areas of poor performance, help nurses make career development plans, and improve their career success levels. This will play a pivotal role in reducing the resignation rate of nurses, improving the enthusiasm of nurses and stabilizing the nursing team, thereby promoting the development of the nursing team and improving the quality of nursing services.

## Data Availability

The datasets generated and analyzed during the current study are not publicly available due the datum involve participant privacy concerns but are available from the corresponding author on reasonable request.
